# Supplementary material for: Combination Therapy with a Bispecific Antibody Targeting the hERG1/β1 Integrin Complex and Gemcitabine in Pancreatic Ductal Adenocarcinoma
Source: Cancers (Basel). 2023 Mar 28;15(7):2013. doi: 10.3390/cancers15072013 (PMC10093586; doi:10.3390/cancers15072013)
Supplement: Supplementary file 1 [file cancers-15-02013-s001.zip › cancers-2231680-supplementary.pdf]

Supplementary information

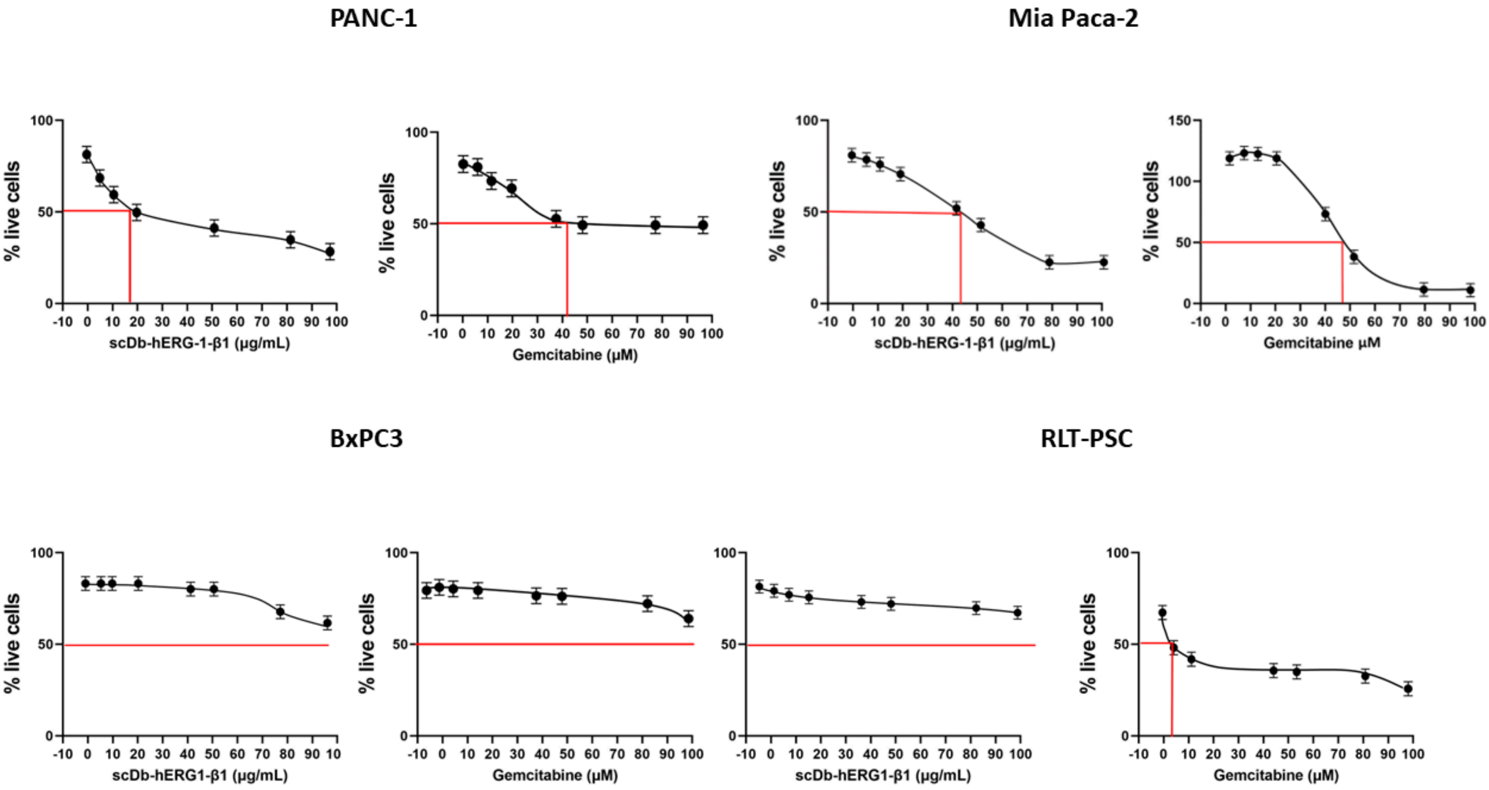

Figure S1: Dose-dependence curves of PANC-1, Mia-Paca2, BxPC3, RLT-PSC treated with different concentrations of scDb-hERG1-β1 and Gemcitabine for 24 hours. The intercept between the black curve and the red line indicates the IC50 value.

	Control	Gemcitabine 25mg/Kg	Gemcitabine 5mg/Kg	scDb-hERG1-β1	**Mean survival time (Days)**
Control	-				67
Gemcitabine 25mg/Kg	0.83	-	-	-	77
Gemcitabine 5mg/Kg	0.22	**0.02**	-	-	59
scDb-hERG1-β1	0.10	0.44	**0.05**	-	79
scDb-hERG1-β1 + Gemcitabine 5mg/Kg	0.33	0.81	0.10	0.71	82

Table S1: Pairwise comparison for Kaplan-Meier survival curve. The p values calculated with Long-Rank test are reported. The last column show mean survival time of all the treatment groups.

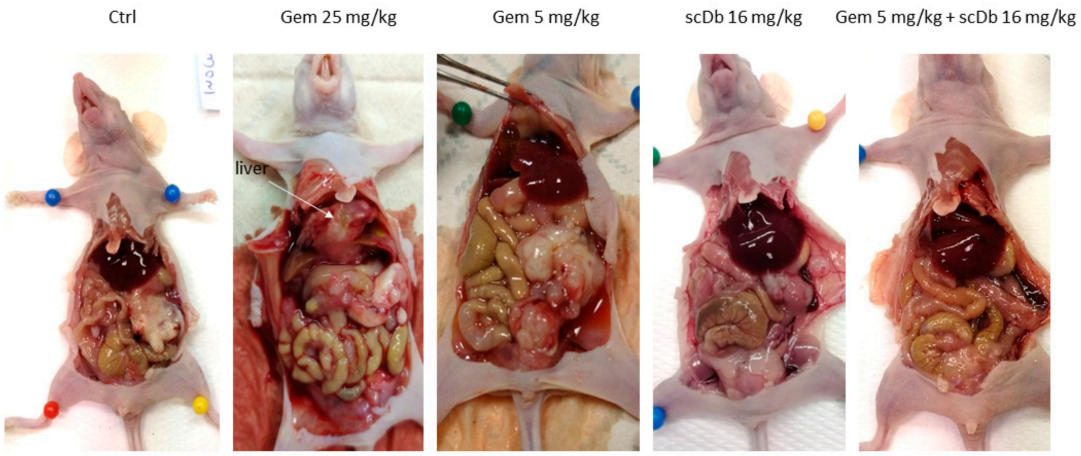

***Figure S2**: Abdominal gross view at the end-point (the day of euthanasia) showed abnormal liver in mice treated with Gemcitabine at the dose of 25 mg/kg, while in the other groups of treatment no abnormalities were observed.*

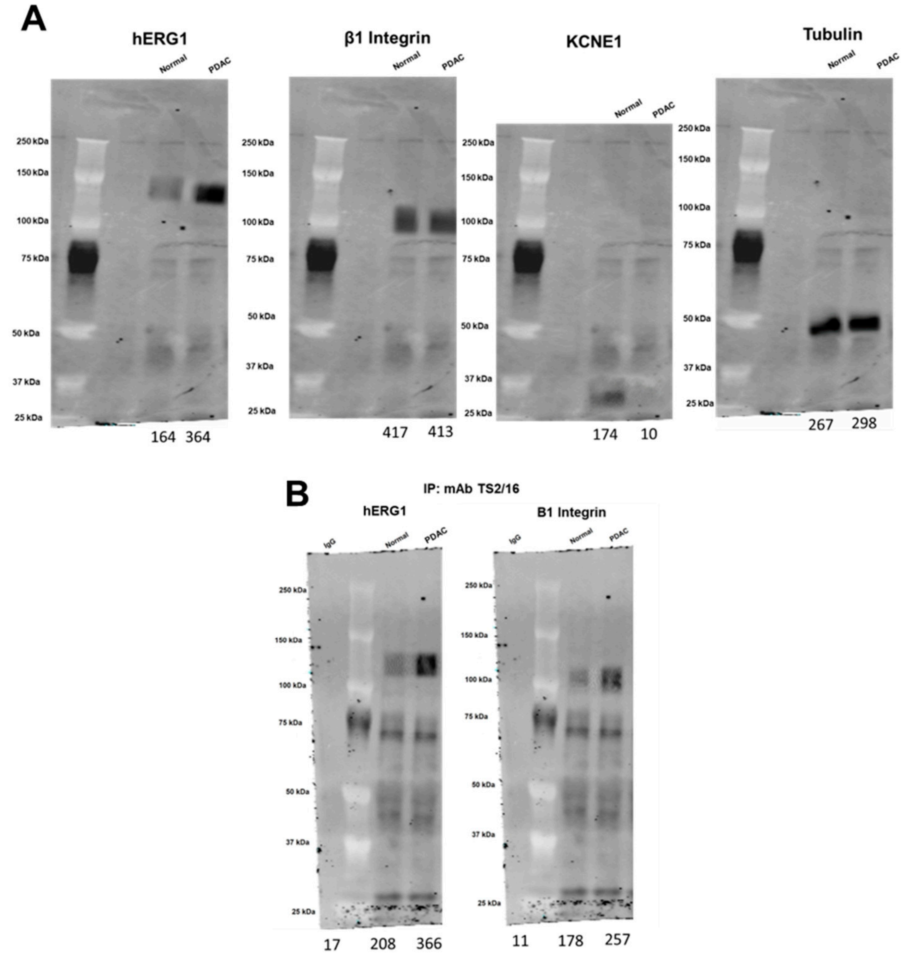

Figure S3: Whole blots of figure 1C (A) and figure 1D (B) with densitometry readings/intensity ratio of each band.
